# Overview of Strategies to Improve Therapy against Tumors Using Natural Killer Cell

**DOI:** 10.1155/2020/8459496

**Published:** 2020-01-21

**Authors:** Chaopin Yang, Yue Li, Yaozhang Yang, Zhiyi Chen

**Affiliations:** ^1^Department of Ultrasound Medicine, Laboratory of Ultrasound Molecular Imaging, The Third Affiliated Hospital of Guangzhou Medical University, Guangzhou 510150, China; ^2^Experimental Center, The Liwan Hospital of the Third Affiliated Hospital of Guangzhou Medical University, Guangzhou 510176, China

## Abstract

NK cells are lymphocytes with antitumor properties and can directly lyse tumor cells in a non-MHC-restricted manner. However, the tumor microenvironment affects the immune function of NK cells, which leads to immune evasion. This may be related to the pathogenesis of some diseases. Therefore, great efforts have been made to improve the immunotherapy effect of natural killer cells. NK cells from different sources can meet different clinical needs, in order to minimize the inhibition of NK cells and maximize the response potential of NK cells, for example, modification of NK cells can increase the number of NK cells in tumor target area, change the direction of NK cells, and improve their targeting ability to malignant cells. Checkpoint blocking is also a promising strategy for NK cells to kill tumor cells. Combination therapy is another strategy for improving antitumor ability, especially in combination with oncolytic viruses and nanomaterials. In this paper, the mechanisms affecting the activity of NK cells were reviewed, and the therapeutic potential of different basic NK cell strategies in tumor therapy was focused on. The main strategies for improving the immune function of NK cells were described, and some new strategies were proposed.

## 1. Introduction

Natural killer (NK) cells are the first line of antitumor lymphocyte cells [1]. They can directly lyse tumor cells in a non-MHC-restricted manner without prior activation or regulate the adaptive immune response with secreting immune regulatory cytokines [2–5]. There are many different factors influencing the NK cell functions. Firstly, it is the source of NK cells. For example, the NK cell line is an “off the shelf” cellular therapeutic, induced pluripotent stem cell-derived natural killer cells (iPSC-NK cells) have the advantages of homogenous and low immunogenicity, and peripheral blood stem cell- (PBSC-) derived NK cells can be gained from patients directly [6–8]. The function of NK cells is regulated by the interactions between receptors on NK cells and ligands on tumor cells, for instance, the activating receptors NK group 2D (NKG2D) receptor can recognize ligands displayed on the surface of tumor cells and improve its cytotoxicity [9]. But the tumor cells also evoluted various ways to escape the immune surveillance. One effective strategy to prevent immune escape is to modify the surface marker of NK cells, such as CAR-NK [10, 11]; the other strategy is to use monoclonal antibodies to block the inhibitory receptor, a promising treatment strategy called checkpoint blockade [12, 13]. The infiltration number of NK cells in tumor site is also a key factor that influences the treatment effect of NK cells. Many strategies were explored to improve the NK cell number in target sites, for instance, genetic modification of NK cells with chemokine receptor targeting tumor cells could improve the tendency to tumor site [14]. The physical methods such as ultrasound-mediated delivery were also involved to improve the NK cell infiltration in tumor site [15, 16]. To fulfil the ability of NK cell-based therapy, oncolytic virus, nanomaterials, and other physical methods were also involved to improve the NK cell therapy [17, 18].

In this paper, the mechanism affecting NK cells' activity was reviewed, and recent advances of innovative approaches based on NK cell therapy were also discussed. Particularly, we focused on studies indicating the therapeutic potential of different NK cell-based strategies for the management of tumor and try to indicate new breakthroughs and trends in the area of NK cell-based therapy.

## 2. The Key Factors in NK Cell Education

The NK cells' function was regulated by the interactions between receptors on NK cells and ligands on tumor cells. The most important receptors on NK cells are major histocompatibility complex, also known as human leukocyte antigens (HLA) in human or Ly49 in mice. In this way, NK cells can sense the downregulation of MHC molecule to mount an effector response to damaged or infected cells in an “altered self” way. Based on whether the NK cell receptors (NKRs) can identify HLA-I or not, there are two predominant superfamilies of NKRs that have been identified.

### 2.1. HLA-I-Reliant Receptors

#### 2.1.1. Killer Immunoglobulin-Like Receptors (KIRs)

**Figure 1:**
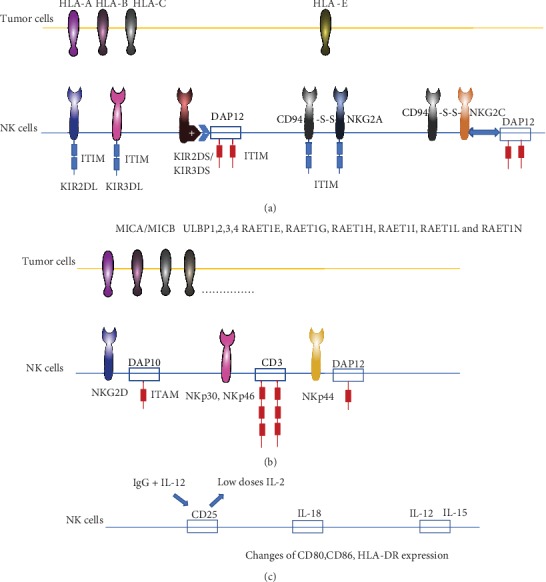
The key factors in NK cell education. (a) HLA-reliant receptors. (b) The activating receptor does not rely on HLA-1. (c) Interleukin improves NK cell response. HLA-1: human leukocyte antigen 1; ITIM: immunoreceptor tyrosine-based inhibitory motif; ITAM: immunoreceptor tyrosine-based activation motif.

The activating and inhibitory KIR receptors control the development and function of NK cells adjusting to the tumor microenvironment immunity [19]. The interactions between KIRs and their HLA class I ligands in humans (Ly49 in mice) mediate NK cell self-tolerance or facilitating cytotoxicity against transformed cells. KIRs can bind to HLA-A, HLA-B, and HLA-C molecules and signal through long intraplasmatic tails with two immunoreceptor tyrosine-based inhibition motifs (ITIMs). In the absence of infection, inhibitory HLA-KIR signals dominate and protect cells from NK cell-mediated lysis [20], while KIR2DL and KIR3DL, binding ITIMs in intracytoplasmic, can inhibit the NK cells from lysis. For KIR2DS and KIR3DS, the DAP-12 can bind to immunoreceptor tyrosine-based activation motif (ITAM), which can activate signal and boost the NK cells to recognize tumor cells [21] (Figure 1(a)).

#### 2.1.2. Killer Lectin-Like Receptors (KLRs)

Separate from KIRs, other conserved MHC-binding receptors including killer lectin-like receptors confer additional diversity in NK education and protection from autoreactivity. HLA-E can be recognized by CD94/NKG2A and CD94/NKG2C for inhibition or activation, respectively. CD94/NKG2A is an example of KLR and has a similar structure that contains a ligand-binding domain resembling that of a C-type lectin. The inhibitory CD94/NKG2A NK cell repertoires have been classified based on their reliance on KIR or CD94/NKG2A for recognition of self-HLA [22]. CD94/NKG2C functions as an activating receptor by associating with the DAP12 signaling adapter [23]. C-type lectin-activating receptors CD94/NKG2C receptors of NK and their activated counterparts have short cytoplasmic tails with ITAMs. The other one is C-type lectin receptors that bind to nonclassical class I MHC molecules or “class I-like” molecules.

### 2.2. The Activating Receptor Does Not Rely on HLA-I

#### 2.2.1. Natural Killer Group 2D (NKG2D)

There are some receptors on the surface of NK cells which do not rely on HLA-I, such as the activating receptors NKG2D, a C-type lectin receptor, which can bind stress-inducible ligands including the MHC class I chain-related (MIC) peptides (MICA and MICB), and the human cytomegalovirus UL16 binding proteins (ULBPs) [24, 25]. The NK cells with NKG2D expression can detect and eliminate cells that have undergone “stress”, showing its promising application in the treatment of infectious disease, cancer, and autoimmune disease [26]. However, advanced cancers frequently lost cell surface-bound MICA and MICB by proteolytic shedding and escape NK cell immune mechanism. When researchers specifically adopt small molecule inhibitors to block MICA and MICB, it can reactivate NK cells' antitumor immunity [27]. Some researchers also demonstrated that the circulating adoptive NK cells in patients with metastatic melanoma or renal cell carcinoma did not mediate tumor regression because of its significant low levels of NKG2D expression. When reacted with IL-2 in vitro, the NK cells with improved NKG2D expression could lyse tumor cells in vitro again [28]. Recent research also showed that CD4+CD25+ regulatory T cells (Treg) inhibited NKG2D-mediated NK cell cytotoxicity in vitro [29]. Therefore, it is important to figure out the exact mechanism of NKG2D signal pathway in NK cells.

**Figure 2:**
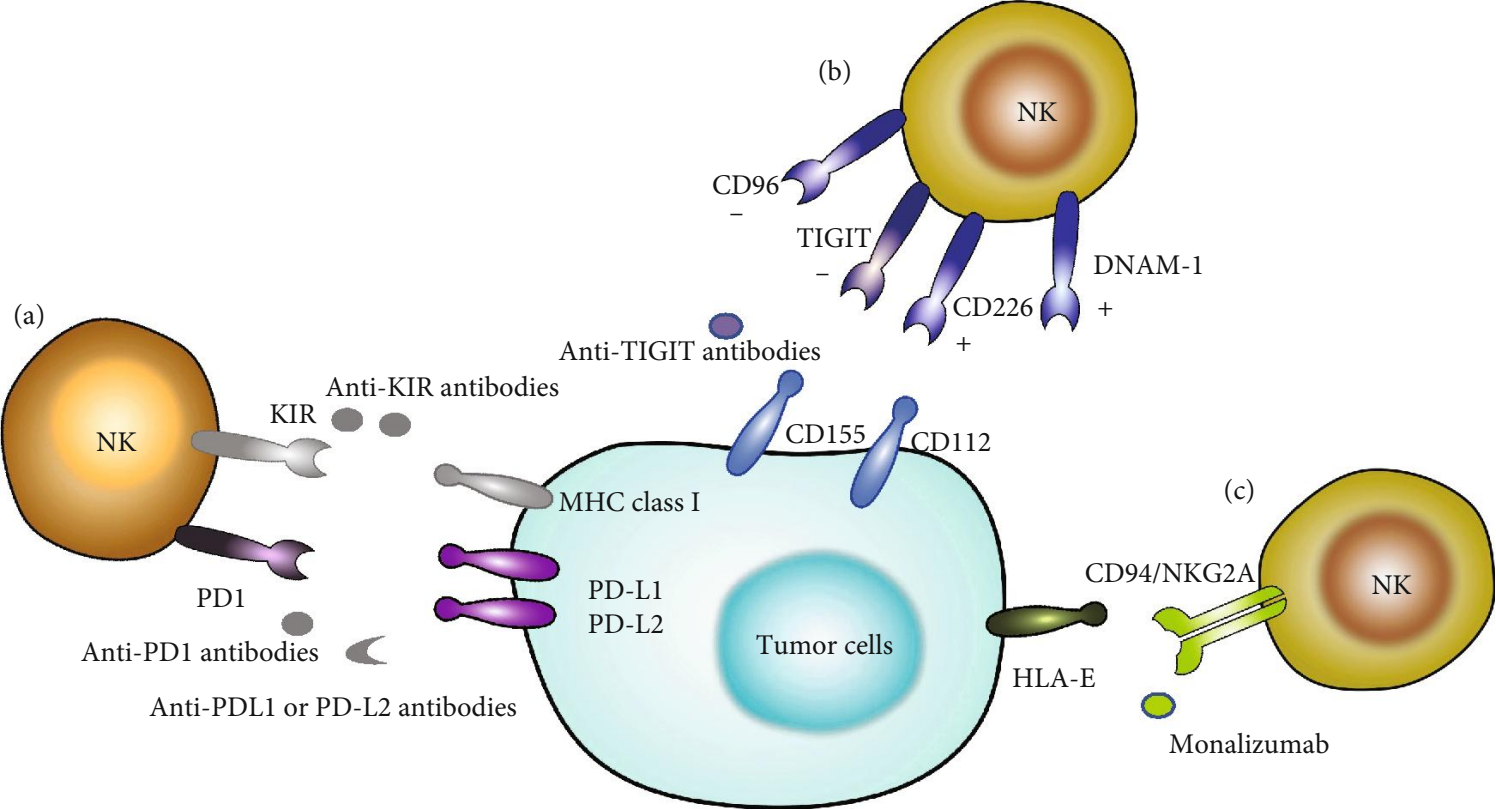
Checkpoint blockade therapy based on NK cells. (a) Using anti-PD1, anti-PDL1, anti-PDL2, or anti-KIR antibodies can release the inhibitory signal for NK cells. (b) TIGIT competes with immune activator receptor CD226 or DNAM-1 for the same set of ligands: CD155 and CD112; blocking CD96 is a promising therapeutic role combined with TIGIT. (c) CD94/NKG2A is an inhibitory receptor expressed by NK cells and T cells that recognizes HLA-E.

The most well-characterized activating NKRs are currently not well defined. NKG2D encoded by a highly conserved gene (KLRK1) with limited polymorphism is an activating receptor expressed on the surface of NK cells, which can recognize an extensive repertoire of ligands, encoded by at least 8 genes in humans (MICA, MICB, RAET1E, RAET1G, RAET1H, RAET1I, RAET1L, and RAET1N) (Figure 2(b)). The NKG2D pathway serves a mechanism for the immune system to detect and eliminate cells that have undergone “stress.” NKG2D provides an attractive target for therapeutics in the treatment of infectious disease, cancer, and autoimmune disease. But a more recent investigation observed that Treg inhibited NKG2D-mediated NK cell cytotoxicity in vitro.

#### 2.2.2. Natural Cytotoxicity Receptors (NCRs) NKp46, NKp30, and NKp44

The NK cells secrete cytokines such as IFN-*γ* and TNF*α* via activating the natural cytotoxicity receptors which consisted of three receptors (NKp46, NKp30, and NKp44) [30]. NKp46 and NKp30 are expressed on the cell membrane of human peripheral blood NK cells, most of them are CD56 dim NK cells, while NKp44 is expressed on IL-2-activated NK cells and CD56 bright NK cells [31]. And ligands are systemically summarized in Kruse study [32].

### 2.3. Interleukin Improves NK Cell Response

Interleukin is an important immunostimulatory cytokine for immune cells including NK cells. IL-2 can enhance NK cell antitumor effects and systemic use along with NK cell-based treatment. It is reported that CD25 on NK cells, a component of the high-affinity IL-2R, can promote NK cell activation in response to low doses of IL-2. Some studies demonstrated that the use of both IgG and IL-12 can improve CD25 expression and further promote NK cell antitumor activity in response to low-dose IL-2 (10 pg/ml) [33]. For NK92 cells, tethering IL-2 to its receptor IL2R*β* can enhance antitumor activity and expansion [34]. Some other studies also demonstrated that when adding both IL-2 and IL-18, the NK cell clusters were observed earlier and NK cells promote the expansion of 56 folds on average on day 10 compared with stimulation with IL-2 or IL-8 alone. The potential mechanism is that IL-18 not only promoted the expansion of NK cells but also changed the phenotype of NK cells. It was observed that IL-18 enhanced the expression of CD80, CD86, and HLA-DR on NK cells, suggesting that IL-18 conferred NK cells an APC-like phenotype [35]. The NK cell-based immunity can also be augmented with other cytokines, such as IL12 and IL15 which can prompt peripheral NK cell tissue homing [36]. Some researchers demonstrated that ALT-803, an IL-15 superagonist complex alone, could also enhance the function of both normal and ovarian cancer patient-derived NK cells by increasing cytotoxicity and cytokine production [37] (Figure 1(c)).

## 3. Sources of NK Cells

There are various sources which could be used for NK cell immunotherapy, such as peripheral blood (PB), umbilical cord blood (UCB), bone marrow (BM), human embryonic stem cells (hESCs), mononuclear cells, induced pluripotent stem cell-derived NK cells (iPSC-NK cells), cord blood-derived NK cells, and NK cell lines [38, 39] (Table 1). Among them, PBNK cells have characteristics of safety, convenience, and strong killer ability against tumor cells [40]. However, NK cells from tumor patients may not expand sufficiently or NK cells with antitumor ability were rarely found. And a suitable donor providing allogeneic NK cells is evaluated strictly and not easy to find. Furthermore, it is time-consuming and costly during NK cell isolation from peripheral blood, as it is always mixed with monocytes and other blood cells. Both autologous and allogeneic NK cells have been proven to be safe and tolerable in adoptive cell therapy [41, 42]. And some studies demonstrated that allogeneic NK cells exhibit better antitumor efficacy than autologous NK cells [43] because the autologous NK cells are potentially inhibited by self MHC class-I molecules on cancer cells. Umbilical cord blood (UCB) is a promising source of allogeneic NK cells which is an off-the-shelf frozen product to generate large numbers of highly functional NK cells from frozen UCB unites ex vivo [44]. iPSC-NK cells also have the characteristics to be banked and stored. And it has been established successfully to obtain large numbers of homogenous NK cells [45]. For acquiring NK cells with better antitumor ability, some researchers compared artificial antigen-presenting cells (aAPCs) expanded PB-NK and iPSC-NK cells with overnight-activated PB-NK cells. The researches demonstrated that aAPCs expanded PB-NK and iPSC-NK cells have better antitumor effect in vivo [46]. Compared with other sources of NK cells, NK cell lines such as NK-92 cells have the obvious advantage of being “off-the-shelf.” The NK-92 cells have also entered clinical trials successfully [47]. And high intravenous doses of up to 10^10^ cells with some beneficial effect reported in melanoma and lung cancer patients have shown to be safe.

## 4. Modification of NK Cells

### 4.1. Chimeric Antigen Receptors

The tumor cells can escape immune surveillance by NK cells. Thus, it is necessary to modify NK cells with chimeric antigen receptors (CARs) to redirect their antitumor ability against resistant tumor cells [48]. To date, NK cells derived from different sources are modified with various CARs to redirect against different cancer antigens such as CD19, CD20, CD244, and HER2. For example, NK-92 cells were engineered to express a variety of CARs specific against tumor-associated proteins (e.g., CD38, CD19, CD20, epithelial cell adhesion molecule (EPCAM), disialoganglioside (GD2), epidermal growth factor receptor variant III (EGFRvIII), and ErbB2 (HER2)), which rendered NK-92 cells cytotoxic against otherwise resistant hematologic and solid malignancies [49, 50].

To avoid side effects of on targets/off tumor side because some normal tissue also express cancer antigens, some efforts insert suicide gene into CAR in order to control this potential cytotoxicity. Liu et al. transduced cord blood-derived NK cells with a retroviral vector incorporating the genes for CAR-CD19, IL-15, and inducible caspase-9-based suicide gene (Ic9), and it demonstrated efficient killing of CD19-expressing cell lines and primary leukemia cells in vitro, with dramatic prolongation of survival in a xenograft Raji lymphoma murine model [51]. Clinical studies have indicated that CD19, CD7, and CD33 CAR-modified NK cells may be a good treatment option for lymphoma and leukemia [52, 53]. Just not long ago, Fate Therapeutics announced that the Food and Drug Administration has approved its research-based new drug (IND) application for FT596. FT596 is derived from the cloned primary induced pluripotent stem cell (iPSC) line iPSC-derived CAR-NK cells, which exhibit similar activity to CAR-T cells but are less toxic. CAR-modified natural killer (NK) cells derived from human induced pluripotent stem cells (iPSCs) (NK-CAR-iPSC-NK cells) in mouse ovaries enhanced antitumor activity in cancer models [54]. And this iPSC-derived CAR-NK therapy is approved for clinical use to address CAR-T recurrence and cost challenges.

Most CARs are derived from CAR-T and directly used to modify NK cell. These strategies omit the difference of intracellular mechanism between NK cells and T cells. Li optimized the CAR specially for NK cell, utilizing NKG2D, a type 2 transmembrane-anchored C-type lectin-like protein; the 2B4 costimulatory domain and the CD3z signaling domain have a better result compared with the specifically target cancer cells in an antigen-specific manner to improve survival in an ovarian cancer xenograft model [54].

### 4.2. Chemokine Receptor

Adoptive NK cell transfer is a promising method for cancer treatment, but only works in a few patients with hematological malignancies. One reason of the poor effect against solid tumor is that circulating NK cells are difficult to enter into tumor. Chemokine plays a significant role in regulation of the migration of leukocytes and it is feasible to improve NK cells homing through genetic expression of corresponding chemokine receptors. Some researchers modified human primary NK cells with CXCR2 to improve their ability to specifically migrate to renal cell carcinoma tumor which readily secreted CCR2 ligands. And CXCR2-transduced NK cells have increased adhesion with renal cells and prompt enhanced killing ability [55–57]. Some researchers also modified NK cells with chemokine receptor CCR7 via trogocytosis, a strategy to maintain the K562-based “donor” cell line expressing CCR7 and NK cells together. Through this process, the NK cells which can acquire CCR7 receptors via trogocytosis will move toward CCL19 and CCL21, then will be homing to the lymph node [58].

### 4.3. Checkpoint Blockade

Recent advances in immune checkpoint blockade (ICB) have revolutionized cancer treatment [59, 60]. The 2018 Nobel Prize in Physiology and Medicine was also awarded to the discoverers of cytotoxic T lymphocyte-associated protein 4 (CTLA4) and programmed cell death 1 (PD1) [61, 62]. Until now, six antibodies targeting immune checkpoint pathways have been approved for clinical use: ipilimumab (anti-CTLA-4), nivolumab (anti-PD-1), pembrolizumab (anti-PD-1), atezolizumab (anti-PD-L1), durvalumab (anti-PD-L1), and avelumab (anti-PD-L1). The mechanism of checkpoint blockade therapy was using monoclonal antibodies (mAbs) to activate immune cells through blocking the inhibitory receptors with ligands expressed on tumor cells. However, much attention is focusing on T cell immunotherapy. Considering NK cells have an important role in innate immunity, the roles of traditional and novel checkpoint blockades in NK cell-based immunotherapy are needed to be identified (Figure 2) [63].

#### 4.3.1. PD-1

Blocking programmed death 1 (PD-1) showed its promising prospect to improve T cell functions and prevent tumor from immune responses. The expression/function of PD-1 on human NK cells was also explored recently. It was reported that CD16-KHYG-1 overexpress PD-1. And PD-1 blockade can block lytic granule polarization in NK cells, accomplished with function impairment of integrin outside-in signal pathway, which indicated that a novel mechanism of how PD-1 inhibition will disrupt NK cell function [64]. Some researchers also identify an increased number of PD-1 (bright) NK cells in the ascites of a cohort of patients with ovarian carcinoma. And PD-1 blockade strategy can restore part of PD-1 (bright) NK cell function [65]. Some studies support the use of cetuximab, which mediated antibody-dependent cytotoxicity (ADCC) against epidermal growth factor receptor- (EGFR-) overexpressing tumor cells, and PD-1 blockade can reverse NK cell dysfunction in head and neck cancer [66]. Furthermore, the safety of PD-1 blockade immunotherapy was confirmed in patients with different cancers.

#### 4.3.2. KIRs

The KIRs presented on NK cell surface recognize MHC class I on tumor cells to maintain a “self-tolerance” condition. The anti-KIR antibodies can reactivate NK cells to kill tumor cells again. Lirilumab, an anti-KIR antibody, has shown a therapeutic effect in mice models of acute myeloid leukemia (AML) and B cell lymphoma. And anti-KIR monotherapy in patients with different cancers has been proven to be safe [67]. And combination of anti-KIR antibody IPH2101 and lenalidomide in phase I trial was also evaluated. But some reports showed the failure of lirilumab in treatment of multiple myeloma in phase II clinical trials [68].

#### 4.3.3. TIGIT

T cell immunoglobulin and immunoreceptor tyrosine (TIGIT), an immunoglobulin superfamily receptor, presented on both NK and T cells and can be characterized to recognize CD155 [69, 70]. Many researches showed that inhibitory receptors TIGIT, CD96, or activating receptors CD226 (also called DNAM-1) compete with the same receptors CD155 (poliovirus receptor) or CD112 (Nectin-2 or PVRL2), such competence regulate the NK cell functions [71–74].

A research reported that blocking CD96 directly or affecting CD96 through blocking TGF-*β*1 can break the balance and reverse NK cell exhaustion of liver patients, CD96 may be a promising inhibitory receptor which could combine with TIGIT in the future [75]. Recent studies also demonstrated that TIGIT blockade not only prevents NK cell exhaustion but also promotes T cell immunotherapy effects when NK cells existed. And the combination of TIGIT and PD-L1 blockade therapy has a better treatment effect in B16 pulmonary metastasis model [76]. The TIGIT blockade combined with anti-PD-1/PD-L1 antibodies has been proved to have synergy in preclinical models. And ongoing TIGIT blockade in phase I trials will answer whether TIGIT will work as well as PD-1/PD-L1 blockade. TIGIT is an immune checkpoint that inhibits activation of T cells and NK cells. A recent study showed that blockade of the checkpoint receptor TIGIT prevents NK cell exhaustion and elicits potent tumor-specific T cell immunity in an NK cell-dependent manner. TIGIT competes with immune activator receptor CD226 or DNAX accessory molecule-1 (DNAM-1) for the same set of ligands: CD155 (PVR or poliovirus receptor) and CD112 (Nectin-2 or poliovirus receptor-related 2 (PVRL2)) [71].

Some other studies demonstrated that CD16+ NK cells highly express TIGIT. When using anti-TIGIT or anti-CD112R, respectively, or together, human NK cells will trigger antibody-dependent cellular cytotoxicity (ADCC) via trastuzumab stimulation. Furthermore, this study indicates that poliovirus-like molecules are promising target in regulating NK cell function [77]. CD96, CD226 (DNAM-1), and TIGIT belong to an emerging family of receptors that interact with nectin and nectin-like proteins. CD226 activates NK cell-mediated cytotoxicity, whereas TIGIT reportedly counterbalances CD226. In contrast, the role of CD96, which shares the ligand CD155 with CD226 and TIGIT, has remained unclear. Some researchers found that CD96 competed with CD226 for CD155 binding and limited NK cell function by direct inhibition. Blocking CD96 may have applications in pathologies in which NK cells are important [74].

#### 4.3.4. NKG2A

Despite the interactions between KIR and HLA-I control NK cell activation or inhibition, the activating receptors NKG2C and inhibitory NKG2A were also interacted with HLA-E on tumor cells [78]. Some researchers found NK cells expressing high NKG2A expression were exhausted and indicated a poor prognosis [79]. Using monalizumab, a monoclonal antibody which can block NKG2A, can improve NK cell cytotoxicity and ADCC [80, 81], especially in preclinical models [82], and some were under clinical trial [83]. Some researchers reported that IL-10 can enhance NKG2A expression. When blocking IL-10, the NKG2A expression on NK cells will decrease. It is reasonable that a higher HLA-E expression on tumor cells will destroy NK cell function. But some researchers reported that HLA-E was lowly expressed on U266 cells but upregulated *in vivo* in RAG-2(-/-) *γ*c (-/-) mice. These researches indicated that HLA-E levels in vitro cannot not be an indicator *in vivo*. And further study demonstrated that both NKG2A-negative and KIR-ligand-mismatched NK cells are much powerful application [84].

#### 4.3.5. CIS

CIS protein is a class of intracellular regulatory proteins known for its inhibition of cytokine signaling pathways. CIS protein has a central SH2 domain that binds to phosphorylated tyrosine motifs. In addition, the c-terminal of CIS protein has a domain called SOCS, which binds to the E3 ubiquitin ligase complex to facilitate ubiquitination and proteasome-mediated protein degradation. Delconte et al. found that the mutant NK cells were significantly more responsive to proliferation, killing, and activation after IL-15 stimulation than the control group. Transcriptome level detection found that the absence of CIS led to changes in the expression levels of many genes, including some serine proteases or their inhibitors, apoptosis regulators, transcription factors, and deubiquitinating enzymes. Through *in vitro* detection of NK cells, the author found that the absence of CIS could improve the signal strength of IL-15, in which JAK-STAT played an important role. Finally, the study demonstrated that blocking CIS can effectively reduce tumor progression by using lung and breast cancer models in mice [85].

#### 4.3.6. IL-1R8

Interleukin-1 receptor 8 (IL-1R8, also known as SIGIRR or TIR8) is a member of the IL-1 receptor (ILR) family and has different structural and functional characteristics from other ILRs. Its primary role is the downstream signaling pathways and inflammatory responses of ILR, and Toll-like receptors (TLRs) play a negative regulatory role [86]. IL-1R8 is an important checkpoint for NK cell maturation and killing functions and plays a negative regulatory role in the antitumor and antiviral effects of NK cells. Recently, Molgora et al. have conducted in-depth research on the regulation mechanism of NK cells in antitumor and antivirus. The results showed that the interleukin receptor molecule IL-1R8 on the surface of NK cells has a negative regulatory effect on the antitumor and antiviral effects of NK cells [87]. The method of using the genetic engineering method to silence the expression of IL-1R8 molecules can effectively inhibit liver cancer, metastatic liver cancer, and cytomegalovirus infection.

## 5. How to Improve NK Cell Infiltration in Target Sites

**Figure 3:**
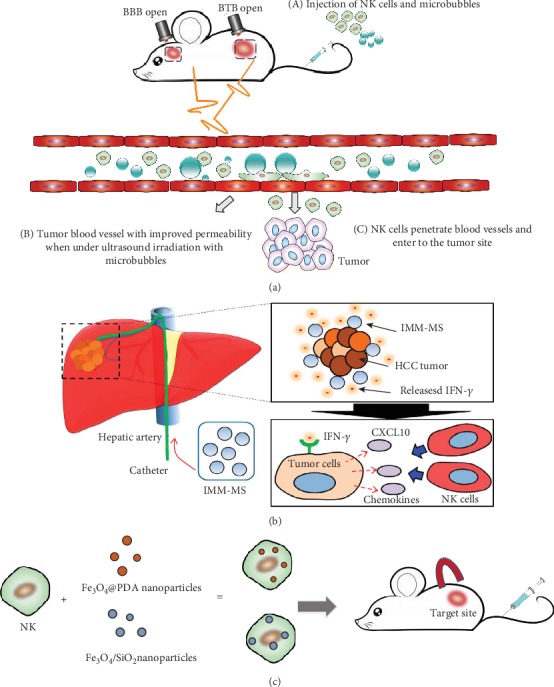
Strategies to improve NK cell infiltration in target sites. (a) Focused ultrasound improved NK cell infiltration in tumor sites. (b) Transcatheter intra-arterial local delivery of chemoattractant encapsulated in polymeric microspheres (reproduced from Ref. [93]). (c) Using magnets can attract NK cells which has uptake Fe_3_O_4_@PDA nanoparticles.

One challenge for NK cell-based immunotherapy is the inadequate homing of adoptive transfer cell therapy [88–90]. Thus, it is urgent to explore novel strategies to improve NK cell infiltration in target sites (Figure 3). Ultrasound-mediated delivery has showed its promising prospect for enhancing the NK cell homing. Blood-brain barrier has been regarded as the main barrier that affected the efficiency of systemic injection of NK cells for brain tumor treatment. Alkins et al. demonstrated that the combination of focused ultrasound (0.33 MPa average peak rarefaction pressure) and intravenous Definity ultrasound contrast can improve NK cell crossing through the BBB. With the interactions between focused ultrasound (FUS) and ultrasound contrast, the number of NK cells in metastatic breast tumor in the brain has a significant increase compared with delivery without BBB disruption. However, Alkins et al. also demonstrated that biweekly treatments with this method have no significant differences compared with the group of NK cells alone. Only adopting an early intensive treatment with targeted NK-92 cells (five times in the first five days and FUS), the tumor will be reduced statistically and further resulted in long-term survival in 50% of subjects [91].

Magnetic field is another useful strategy to increase NK cell recruitment and infiltration in the tumor sites. The key for this strategy is that NK cells have the ability to uptake nanoparticles containing Fe without affecting NK cell viability and cytotoxicity. Some researchers demonstrated that NK-92MI cells uptaking Cy5.5-conjugated Fe_3_O_4_/SiO_2_ core/shell nanoparticles can have a 17-fold enhancement into tumor compared with NK-92MI cells alone via fluorescence microscopy. Wu et al. used polydopamine (PDA) to adhere Fe_3_O_4_ to improve biocompatibility and biodegradability. When putting a tiny magnetic device inside animals, the tumor will grow slowly compared with Fe_3_O_4_@polydopamine nanoparticle group and PBS group and without systemic toxicity in mice [92].

Considering IFN-*γ* can induce CXCL-10 expression and further result in improved NK cell infiltration, Park fabricated MRI visible immunomodulatory microspheres (IMM-MS) composed of IFN-*γ*, iron oxide nanocubes, and PLGA and demonstrated that such IMM-MS can attract NK cells into orthotopic liver tumor xenograft VX2 rabbit model. Transcatheter intra-arterial (IA) local delivery of chemoattractant encapsulated in polymeric microspheres is another strategy to induce efficient NK cell infiltration specifically for liver cancer. An immunomodulatory microsphere (IMM-MS) composed of biodegradable poly (lactide-co-glycolide) (PLGA) as a biocompatible polymer, IONC as an MR contrast agent, and IFN-*γ* as an immunomodulatory protein agent. The deposition of IMM-MS significantly increased NK cell infiltration into the liver tumor site [93, 94].

## 6. Treatment Strategies Combining NK Cells

### 6.1. Drug Delivery

Human immune cell can be used as vehicles for drug delivery or targeting agents because immune cells have the ability to traffic to the tumor or inflammatory sites [95–97]. NK cells with genetic modification have the ability to traffic to different solid tumors with associated ligand expression. NK92 cell with anti-CD19 to target to modification could serve as paclitaxel (PTX) nanoparticle drug delivery to target SKOV3.CD19 tumor subcutaneously in NSG mice. And the combination therapy through NK92 modified with PTX drug nanoparticles has showed better treatment results in both in vitro and in vivo studies [98]. The core technique of using immune cells in drug delivery is knowing how to attach the drug and leukocytes efficiently without affecting NK cells' naïve ability. And it has also been systematically explored, ranging from cell surface functionalization to internalization within leukocytes, which depends on the types of therapeutic delivered, leukocyte targeted, and the site of delivery [99].

**Figure 4:**
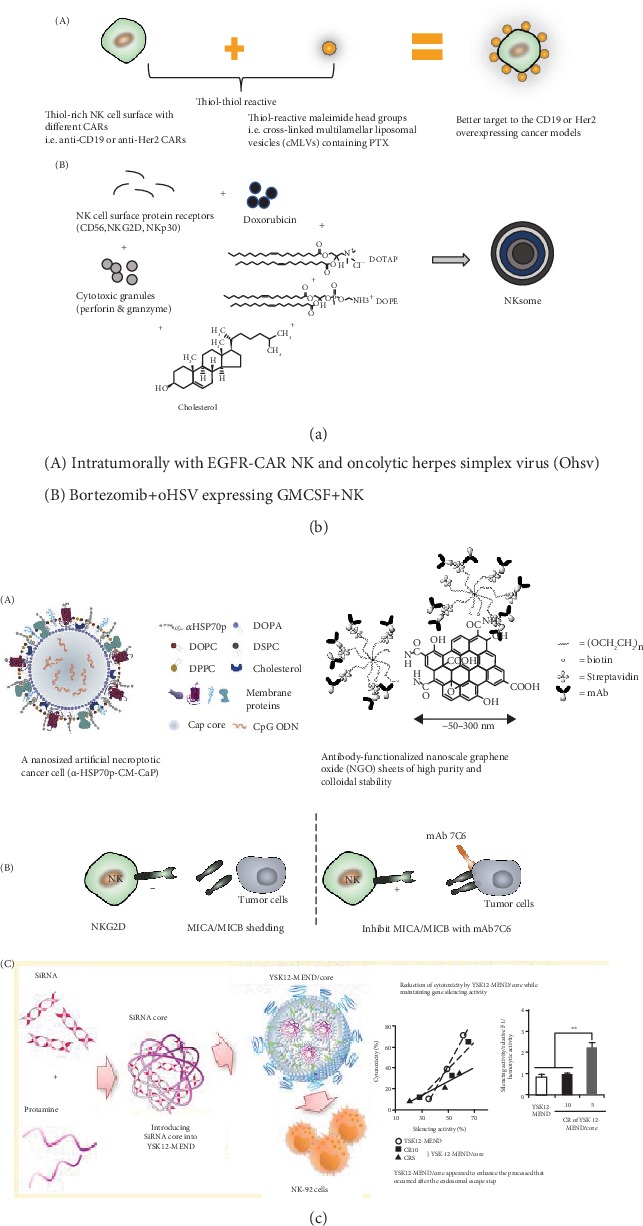
Different combined therapy improved therapeutic effect based on NK cells. (a) The NK cells of genetic modification and the membrane of NK cells can be used for drug delivery. (b) The combination of oncolytic viruses (OVs) and NK cells can improve the antitumor effects. Different combined therapy improved therapeutic effect based on NK cells. (c) The nanoparticles can assist NK cell-based therapy in different aspects. (Photo A of (c) was reproduced from Ref. [104]; photo C of (c) was reproduced from Ref. [107]).

What is more, some novel strategies of using NK cell membranes were also explored. Pitchaimani et al. developed “NKsome” composed of doxorubicin- (DOX-) loaded cationic liposomes and lysis NK-92 cells, which can recognize tumor cells through NK cell markers and further release chemotherapeutic drug DOX [100]. Deng et al. use NK cell membrane-decorated photosensitizer TCPP nanoparticles, which can induce proinflammatory M1 macrophage polarization and further activate effector T cells (CD4+ T cells and CD8+ T cells). A membrane camouflage fusogenic liposomal delivery system called “NKsome” is used for targeted tumor therapy. Activated NK cell membrane with receptor proteins was isolated from the NK-92 cells and extruded with the fusogenic liposome to form NKsomes. And chemotherapeutic drug doxorubicin (DOX) was further encapsulated into the aqueous core of NKsome and has improved its tumor homing ability and antitumor efficacy against MCF-7-induced solid tumor model [101] (Figure 4(a)).

### 6.2. Combination of Oncolytic Virus and NK Cells

Oncolytic virus (OV) therapy is a promising biological therapy, and FDA approved the first oncolytic HSV (oHSV) (T-Vec) used in advanced melanoma in 2015. However, the efficiency of OV therapy was not satisfying. The combined therapy with oncolytic viruses and NK cells was explored. Chen et al. demonstrated that group in EGFR-CAR NK-92 cells for 4 h followed by Ohsv-1 of breast cancer brain metastases mice will have a longer survival compared to monotherapies [102]. Suryadevara et al. combined OVs, proteasome inhibitors, and NK cell immunotherapy, which prolonged survival against glioblastoma (GBM). The mechanism of this triple combined therapy was the proteasome inhibitors bortezomib can increase heat shock protein 90 expression and resulted in OV replication [103]. Furthermore, oHSV and bortezomib will induce necroptosis and stimulate NK cell immunotherapy (Figure 4(b)).

### 6.3. Nanotechnology-Assisted NK Cell Therapy

Biomimetic nanotechnology is promising because it can imitate the physiological process and modulate immune cell functions and stimulate immunotherapy. Kang et al. fabricated a nanosized artificial necroptotic cancer cell (*α*-HSP70p-CM-CaP) vaccine composed of cancer membrane proteins (CM), *α*-helix HSP70 functional peptide (*α*-hsp70p), and CpG to stimulate both natural killer (NK) cells and APC. The tumor-associated antigens (TAA) on cancer cell membrane can trigger adaptive immune system. *α*-HSP70p, a danger-associated molecular pattern (DAMP), can activate both NK cells and DCs via binding with receptor CD49. CpG, an oligodeoxynucleotide, was also used as adjuvant which can stimulate strong host response. This nanosized vaccine (*α*HSP70p-CM-CaP) combined strategies of different antigens were used to acquire maximum antitumor effect. The *α*HSP70p-CM-CaP vaccine combined with anti-PD-1 antibody treatment therapy has provided tumor regression on mice B16OVA melanoma models [104].

To activate NK cells, nanoscale graphene oxide (NGO) with *α*-Hcd16 was synthesized; the cytolytic granule degranulation and IFN-*γ* secretion ability of NK cells were enhanced compared to *α*-Hcd16 alone [105]. The NK cell surface is an important bridge to deliver signals into intracellular level to active NK cells. Thus, the nanomaterials should mimic the biological conditions to interact with NK cell functions [106]. Delivering small interfering RNA (siRNA) into NK cells with high efficiency and low cytotoxicity is a difficult task. Nakamura et al. introduced siRNA core with protamine to decrease the total amount of lipid and further reduce the cytotoxicity. This study demonstrated that the ratio of YSK12-C4/siRNA at 5 has the similar gene silencing efficiency and lower cytotoxicity compared to a multifunctional envelope-type nanodevice (MEND) containing YSK12-C4 [107] (Figure 4(c)).

### 6.4. Bi- and Trispecific Killer Engagers

Recently, bispecific and trispecific antibodies known as killer cell adapters (BiKEs and TriKEs) have been designed to efficiently participate in CD16 and induce antibody-dependent cell-mediated cytotoxicity (ADCC) response. They are designed by recognizing the fusion of the Fv domain of tumor cell antigens with the Fv domain that binds to CD16, with the ultimate goal of linking NK cells to tumor cells. Bispecific monoclonal antibodies have been successfully tested in primary lymphoma and Hodgkin lymphoma [108]. Although many strategies have shown promising success in *in vitro* and *in vivo* preclinical models, some of them are currently being evaluated in clinical trials. BiKEs contain two antibody fragments, one recognizes tumor antigens and the other one targets CD16 on NK cells, which together trigger ADCC. IL-15 is further integrated to create TriKE to drive strength of NK cells [109]. By comparing with BiKEs, TriKEs produced superior NK cytotoxicity and NK cell persistence in *in vivo* xenograft tumor models and were recommended as an effective complement to existing NK delivery protocols. Importantly, TriKEs provide a versatile and cost-effective platform on which to incorporate novel targeting ligands with the potential to stimulate endogenous NK cells, thereby completely avoiding the need for cell transfer, predicting a new generation of immunity therapy.

## 7. NK Cell Tracking

How NK cells function its antitumor response in vivo is still not clear, and it is urgent to develop noninvasive imaging methods to track NK cells in vivo to explore the distribution of adoptive transfer and elucidate mechanism of NK cell-based immunotherapy. Three methods including optical microscopy, PET/SPET, and magnetic resonance imaging (MRI) were used to trace NK cells through labeling NK cells with fluorophores, radiotracers, or paramagnetic nanoparticles, respectively (Table 2).

Tavri et al. reported that a near-infrared dye DiD was employed to track the NK-92 scFv (MOC31)-zeta cells [110]. Lim et al. used anti-CD56 antibody conjugated with quantum dots (QD705) to label NK-92MI cells, which can be traced up to 12 days after intratumoral injection in human melanoma xenograft models [111]. Radiotracers including ^18^FDG and C-methyl iodide were used for PET [110]. SPECT was used to label CAR-NK cells, which proved that targeting NK cells can accumulate more in tumor site compared to nontargeting NK cells [112]. For MRI, superparamagnetic iron oxide (SPIO) and ultrasmall superparamagnetic iron oxide (USPIO) nanoparticles were the most used probe to label NK cells; it is reported that NK cells can retain iron oxide up to 4 days [92, 113].

Conjugation of fluorescence organic dye (Cy5.5) with magnetic nanoparticles (Fe_3_O_4_/SiO_2_) has also been used to visualize the migration of NK-92MI cells to the tumor site controlled by an external magnetic field [114]. PET and SPECT provide high sensitivity for detecting labeled NK cells (in the nanomolar concentration range), high specificity (target to background contrast), quantification of labeled cells and have immediate clinical translation through the use of FDA-approved labeled agents.

## 8. Conclusion

The key strategy for improving NK cell-based immunotherapy is to maximize NK cell reactive potential while minimizing NK cell inhibition. This review tried to give a whole picture of how NK cells were activated or inhibited and showed different strategies to improve the NK cell therapy. Various factors influence the functions of NK cells, the sources, the receptors on NK cell surfaces, the infiltration numbers in tumor sites, and so on. Nowadays, the clinical result of immune cell therapy indicated that adoptively transfer cell therapy and checkpoint blockade are the most promising strategies [115–118].

However, compared to T cell therapy, the mechanisms of NK cells are still needed to be furtherly explored. Luckily, some researches have been proceeded, such as developing the specific CAR for NK cells (NKG2D) rather than using the CAR which is designed for T cells originally. The TIGTI checkpoint blockade was also found to enhance NK cells' killing ability. These results indicated one promising direction is to develop strategies that can precisely regulate the NK cells' function. But the premise of this strategy is based on the full understanding of NK cells' activated and inhibitory functions. Activated NK cells were considered undergoing glycolysis and mitochondrial oxidative phosphorylation (OXPHOS), which is necessary for IFN-*γ* or granzyme B secretion of NK cells. Srebp-dependent regulation and amino acid-controlled c-Myc have been proved necessary for NK cell metabolic and functional responses. But NK cells in solid tumor are always dysfunctional which may be due to the tumor environments [119, 120]. These results indicated novel strategies of stabilizing c-Myc expression in NK cells which may be useful for normal functions of NK cells [121].

With the assistance of oncolytic virus and nanomaterials, the NK cell-based immunotherapy is promising. The oncolytic virus or nanomaterials can be designed to interfere with NK cell inhibition or change the tumor environment favouring NK cells' function. The NK cell in vitro culture is not to maintain the similar ability of NK cells in vivo. Some studies demonstrated that as the culture days prolong, some chemokine receptors of NK cells will be lost. Therefore, when administrating adoptive NK cell therapy, the surface marker expression of NK cells should be paid enough attention to ensure the NK cell ability [122, 123].

Finally, safety problems were important factors that should be considered when utilizing it in clinical studies. For example, targeted / non-targeted tumor toxicity of CAR-engineered NK cells needs to be considered, as for some cancer antigens are also expressed in healthy tissues. Insertion of suicide genes into CAR-modified effectors is one of the strategies to avoid these side effects [124]. However, such genetic modification needs to be sophistically designed and is laborious. Thus, the safety use of NK cells still needs to be further explored.

## Figures and Tables

**Table 1:** The advantages and disadvantages of different sources of NK cells.

Sources of NK cells	Advantages	Disadvantages
Peripheral blood	Safe; conveniently collected; strong ability to kill tumor cells	Low numbers in patients; time-consuming and costly
Umbilical cord blood	Available; off-the-shelf; UCB-derived CD34+ cells have translated to the clinic; frozen for a long time	Only one time to get access to the umbilical cord blood
Human embryonic stem cells or induced pluripotent cell	Homogenous NK cell product; easy to amplify large numbers of NK cells	Need to induce iPSC into NK cells
Bone marrow	From patients	Invasive operation
NK cell line (i.e., NK-92 and NK-92MI)	Off-the-shelf; easy to amplify; lack most inhibitory receptors compared to naïve NK cells	Potential tumorigenicity

**Table 2:** NK cell trafficking methods.

Imaging mode	Probes	NK cell types	Time in which NK cells retain the label	Advantages	Disadvantages
Optical imaging	DiR	Primary NK cells, activated/expanded NK cells, or NK cell lines	The DiR NK cells can be imaged for 8 days.	Little florescence interference; high tissue penetrance; using intravital microscopy	The DiR dye would persist in the membranes even in dead cells.
	Near-infrared dye DiD	NK-92 scFv (MOCC31)-zeta cells targeted to EpCAM antigen in tumors	Long-lasting at 72 h	Easily applicable; fast; inexpensive; and provides highly sensitive noninvasive imaging in preclinical and clinical settings	The absorption and emission wavelength of the probe is less than 65 nm. Many biological tissues are capable of autofluorescence under excitation, interfering with fluorescence analysis and biological imaging of biological samples.
	ESNF	Ex vivo-expanded NK cells	Long-lasting at 72 h using concentrations as low as 0.04 *μ*M	Do not affect NK cell purity, expression levels of surface receptors, or cytotoxic functions	—
	Quantum dots (QD705)	NK-92MI	Up to 12 days post intratumoral injection in tumors	Low toxicity; high quantum yield; color availability; good photostability; large surface-to-volume ratio; surface functionality; and small size	Weakening of nonspecific background
	Ag_2_ Se quantum dots (QDs)	NK-92	—	The emission is 1350 nm, in the second near-infrared window.	—

PET/SPECT	C-methyl iodide	—	Half-life = 20 min for PET as well as with gamma emitting radioisotope ^111^in-oxine	High sensitivity for detecting labeled NK cells; high specificity	Poor spatial resolution (~1-5 mm), limited anatomic information, ionizing radiation, limited duration and number of scanning sessions
	^18^FDG	NK-92-scFv (FRP5)-zeta cells	^18^FDG 2-4 hours to days (^111^In 4-7 days)	Clinically applicable; high specificity and sensitivity	Limited tracer uptake into the cells and loss of ^18^FDG from labeled cells

Magnetic resonance imaging	USPIO	NK-92	2-4 weeks	Without significant adverse effect on the viability of cells; FDA approved	Hardly detecting small cell populations, relative high cost, long scan times, and low specificity; limited sensitivity, limited efficient labeling efficiency

Optical imaging/magnetic resonance imaging	Fluorescence organic dye (Cy5.5) with magnetic nanoparticles (Fe_3_O_4_/SiO_2_)	NK-92MI	—	High specificity	Poor spatial resolution (~1-5 mm); limited anatomic information, ionizing radiation, limited duration and number of scanning sessions

## Abbreviations

NK: Natural killer
MHC: Major histocompatibility complex
HLA: Human leukocyte antigens
NKRs: NK cell receptors
KIRs: Killer immunoglobulin-like receptors
ITIMs: Immunoreceptor tyrosine-based inhibition motifs
ITAM: Immunoreceptor tyrosine-based activation motif
KLRs: Killer lectin-like receptors
NKG2D: Natural killer group 2D
NCRs: Natural cytotoxicity receptors
PB: Peripheral blood
BM: Bone marrow
hESCs: Human embryonic stem cells
iPSC-NK cells: Induced pluripotent stem cell-derived NK cells
aAPCs: Artificial antigen presenting cells
CAR: Chimeric antigen receptors
ICB: Immune checkpoint blockade
PD1: Programmed cell death 1
mAbs: Monoclonal antibodies
ADCC: Antibody-dependent cytotoxicity
EGFR: Epidermal growth factor receptor
AML: Acute myeloid leukemia
TIGIT: T cell immunoglobulin and immunoreceptor tyrosine
PVRL2: Poliovirus receptor-related 2
DAMPs: Danger-associated molecular patterns
iPSC: Induced pluripotent stem cell
FUS: Focused ultrasound.

## References

[1] Guillerey C., Huntington N. D., Smyth M. J. (2016). Targeting natural killer cells in cancer immunotherapy. *Nature Immunology*.

[2] Handgretinger R., Lang P., André M. C. (2016). Exploitation of natural killer cells for the treatment of acute leukemia. *Blood*.

[3] Gao Y., Arkwright P. D., Carter R. (2016). Bone marrow transplantation for MHC class I deficiency corrects T-cell immunity but dissociates natural killer cell repertoire formation from function. *Journal of Allergy and Clinical Immunology*.

[4] Ardolino M., Azimi C. S., Iannello A. (2014). Cytokine therapy reverses NK cell anergy in MHC-deficient tumors. *Journal of Clinical Investigation*.

[5] Zitvogel L., Kroemer G. (2014). Cytokines reinstate NK cell-mediated cancer immunosurveillance. *Journal of Clinical Investigation*.

[6] Suck G., Odendahl M., Nowakowska P. (2016). NK-92: an ‘off-the-shelf therapeutic’ for adoptive natural killer cell-based cancer immunotherapy. *Cancer Immunology, Immunotherapy*.

[7] Saetersmoen M. L., Hammer Q., Valamehr B., Kaufman D. S., Malmberg K. J. (2019). Off-the-shelf cell therapy with induced pluripotent stem cell-derived natural killer cells. *Seminars in Immunopathology*.

[8] Malard F., Labopin M., Chevallier P. (2016). Larger number of invariant natural killer T cells in PBSC allografts correlates with improved GVHD-free and progression-free survival. *Blood*.

[9] Jelenčić V., Šestan M., Kavazović I. (2018). NK cell receptor NKG2D sets activation threshold for the NCR1 receptor early in NK cell development. *Nature Immunology*.

[10] Siegler E. L., Zhu Y., Wang P., Yang L. (2018). Off-the-shelf CAR-NK cells for cancer immunotherapy. *Cell Stem Cell*.

[11] (2017). Equipping NK cells with CARs. *Cancer Discovery*.

[12] (2018). NK cells respond to checkpoint blockade. *Cancer Discovery*.

[13] Sun H., Huang Q., Huang M. (2019). Human CD96 correlates to Natural Killer cell exhaustion and predicts the prognosis of human hepatocellular carcinoma. *Hepatology*.

[14] Ponzetta A., Benigni G., Antonangeli F. (2015). Multiple myeloma impairs bone marrow localization of effector natural killer cells by altering the chemokine microenvironment. *Cancer Research*.

[15] Alkins R., Burgess A., Ganguly M. (2013). Focused ultrasound delivers targeted immune cells to metastatic brain tumors. *Cancer Research*.

[16] Sta Maria N. S., Barnes S. R., Weist M. R., Colcher D., Raubitschek A. A., Jacobs R. E. (2015). Low dose focused ultrasound induces enhanced tumor accumulation of natural killer cells. *PLoS One*.

[17] Wang S., Liu H., Xin J. (2019). Chlorin-based photoactivable galectin-3-inhibitor nanoliposome for enhanced photodynamic therapy and NK cell-related immunity in melanoma. *ACS Applied Materials & Interfaces*.

[18] Wang H., Mooney D. J. (2018). Biomaterial-assisted targeted modulation of immune cells in cancer treatment. *Nature Materials*.

[19] Barani S., Khademi B., Ashouri E., Ghaderi A. (2018). *KIR2DS1, 2DS5, 3DS1 and KIR2DL5* are associated with the risk of head and neck squamous cell carcinoma in Iranians. *Human Immunology*.

[20] Rehermann B. (2016). Peptide-dependent HLA-KIR-mediated regulation of NK cell function. *Journal of Hepatology*.

[21] van der Ploeg K., Chang C., Ivarsson M. A., Moffett A., Wills M. R., Trowsdale J. (2017). Modulation of human leukocyte antigen-C by human cytomegalovirus stimulates KIR2DS1 recognition by natural killer cells. *Frontiers in Immunology*.

[22] Boudreau J. E., Hsu K. C. (2018). Natural killer cell education and the response to infection and cancer therapy: stay tuned. *Trends in Immunology*.

[23] Kaiser B. K., Pizarro J. C., Kerns J., Strong R. K. (2008). Structural basis for NKG2A/CD94 recognition of HLA-E. *Proceedings of the National Academy of Sciences*.

[24] Koch J., Steinle A., Watzl C., Mandelboim O. (2013). Activating natural cytotoxicity receptors of natural killer cells in cancer and infection. *Trends Immunol*.

[25] Pahl J., Cerwenka A. (2017). Tricking the balance: NK cells in anti-cancer immunity. *Immunobiology*.

[26] Lanier L. L. (2015). NKG2D receptor and its ligands in host defense. *Cancer Immunology Research*.

[27] de Andrade L. F., Tay R. E., Pan D. (2018). Antibody-mediated inhibition of MICA and MICB shedding promotes NK cell-driven tumor immunity. *Science*.

[28] Parkhurst M. R., Riley J. P., Dudley M. E., Rosenberg S. A. (2011). Adoptive transfer of autologous natural killer cells leads to high levels of circulating natural killer cells but does not mediate tumor regression. *Clinical Cancer Research*.

[29] Smyth M. J., Teng M. W. L., Swann J., Kyparissoudis K., Godfrey D. I., Hayakawa Y. (2006). CD4+CD25+ T regulatory cells suppress NK cell-mediated immunotherapy of cancer. *The Journal of Immunology*.

[30] Kruse P. H., Matta J., Ugolini S., Vivier E. (2014). Natural cytotoxicity receptors and their ligands. *Immunology & Cell Biology*.

[31] Mattiola I., Pesant M., Tentorio P. F. (2015). Priming of human resting NK cells by autologous M1 macrophages via the engagement of IL-1*β*, IFN-*β*, and IL-15 pathways. *The Journal of Immunology*.

[32] Shemesh A., Brusilovsky M., Kundu K., Ottolenghi A., Campbell K. S., Porgador A. (2018). Splice variants of human natural cytotoxicity receptors: novel innate immune checkpoints. *Cancer Immunology, Immunotherapy*.

[33] Duggan M. C., Campbell A. R., McMichael E. L. (2018). Co-stimulation of the fc receptor and interleukin-12 receptor on human natural killer cells leads to increased expression of cd25. *Oncoimmunology*.

[34] Vivier E., Raulet D. H., Moretta A. (2011). Innate or adaptive immunity? The example of natural killer cells. *Science*.

[35] Senju H., Kumagai A., Nakamura Y. (2018). Effect of IL-18 on the expansion and phenotype of human natural killer cells: application to cancer immunotherapy. *International Journal of Biological Sciences*.

[36] Hydes T., Noll A., Salinas-Riester G. (2018). IL-12 and IL-15 induce the expression of CXCR6 and CD49a on peripheral natural killer cells. *Immunity, Inflammation and Disease*.

[37] Felices M., Chu S., Kodal B. (2017). IL-15 super-agonist (ALT-803) enhances natural killer (NK) cell function against ovarian cancer. *Gynecologic Oncology*.

[38] Bock A. M., Knorr D., Kaufman D. S. (2013). Development, expansion, and *in vivo* monitoring of human NK cells from human embryonic stem cells (hESCs) and and induced pluripotent stem cells (iPSCs). *Journal of Visualized Experiments*.

[39] Fang F., Xiao W., Tian Z. (2018). Challenges of NK cell-based immunotherapy in the new era. *Frontiers of Medicine*.

[40] Shoae-Hassani A., Behfar M., Mortazavi-Tabatabaei S. A., Ai J., Mohseni R., Hamidieh A. A. (2017). Natural killer cells from the subcutaneous adipose tissue underexpress the NKp30 and NKp44 in obese persons and are less active against major histocompatibility complex class I non-expressing neoplastic cells. *Frontiers in Immunology*.

[41] Veluchamy J. P., Kok N., van der Vliet H. J., Verheul H. M. W., de Gruijl T. D., Spanholtz J. (2017). The rise of allogeneic natural killer cells as a platform for cancer immunotherapy: recent innovations and future developments. *Frontiers in Immunology*.

[42] Martin-Antonio B., Sune G., Perez-Amill L., Castella M., Urbano-Ispizua A. (2017). Natural killer cells: angels and devils for immunotherapy. *International Journal of Molecular Sciences*.

[43] Lim O., Jung M. Y., Hwang Y. K., Shin E. C. (2015). Present and future of allogeneic natural killer cell therapy. *Frontiers in Immunology*.

[44] Veluchamy J. P., Lopez-Lastra S., Spanholtz J. (2017). In vivo efficacy of umbilical cord blood stem cell-derived NK cells in the treatment of metastatic colorectal cancer. *Frontiers in Immunology*.

[45] Zhu H., Kaufman D. S. (2019). An improved method to produce clinical-scale natural killer cells from human pluripotent stem cells. *Methods in Molecular Biology*.

[46] Hermanson D. L., Bendzick L., Pribyl L. (2016). Induced pluripotent stem cell-derived natural killer cells for treatment of ovarian cancer. *Stem Cells*.

[47] Tonn T., Schwabe D., Klingemann H. G. (2013). Treatment of patients with advanced cancer with the natural killer cell line NK-92. *Cytotherapy*.

[48] Glienke W., Esser R., Priesner C. (2015). Advantages and applications of CAR-expressing natural killer cells. *Frontiers in Pharmacology*.

[49] Oelsner S., Friede M. E., Zhang C. (2017). Continuously expanding CAR NK-92 cells display selective cytotoxicity against B-cell leukemia and lymphoma. *Cytotherapy*.

[50] Nowakowska P., Romanski A., Miller N. (2018). Clinical grade manufacturing of genetically modified, CAR-expressing NK-92 cells for the treatment of ErbB2-positive malignancies. *Cancer Immunology, Immunotherapy*.

[51] Liu E., Tong Y., Dotti G. (2018). Cord blood NK cells engineered to express IL-15 and a CD19-targeted CAR show long-term persistence and potent antitumor activity. *Leukemia*.

[52] Romanski A., Uherek C., Bug G. (2016). CD19-CAR engineered NK-92 cells are sufficient to overcome NK cell resistance in B-cell malignancies. *Journal of Cellular and Molecular Medicine*.

[53] Tang X., Yang L., Li Z. (2018). First-in-man clinical trial of CAR NK-92 cells: safety test of CD33-CAR NK-92 cells in patients with relapsed and refractory acute myeloid leukemia. *American Journal of Cancer Research*.

[54] Li Y., Hermanson D. L., Moriarity B. S., Kaufman D. S. (2018). Human iPSC-derived natural killer cells engineered with chimeric antigen receptors enhance anti-tumor activity. *Cell Stem Cell*.

[55] Kremer V., Ligtenberg M. A., Zendehdel R. (2017). Genetic engineering of human NK cells to express CXCR2 improves migration to renal cell carcinoma. *Journal for ImmunoTherapy of Cancer*.

[56] Bayer A. L., Pugliese A., Malek T. R. (2013). The IL-2/IL-2R system: from basic science to therapeutic applications to enhance immune regulation. *Immunologic Research*.

[57] Yamin R., Lecker L. S. M., Weisblum Y. (2016). HCMV vCXCL1 binds several chemokine receptors and preferentially attracts neutrophils over NK cells by interacting with CXCR2. *Cell Reports*.

[58] Somanchi S. S., Somanchi A., Cooper L. J. N., Lee D. A. (2012). Engineering lymph node homing of ex vivo-expanded human natural killer cells via trogocytosis of the chemokine receptor CCR7. *Blood*.

[59] Tang J., Yu J. X., Hubbard-Lucey V. M., Neftelinov S. T., Hodge J. P., Lin Y. (2018). The clinical trial landscape for PD1/PDL1 immune checkpoint inhibitors. *Nature Reviews Drug Discovery*.

[60] Meng X., Liu X., Guo X. (2018). FBXO38 mediates PD-1 ubiquitination and regulates anti-tumour immunity of T cells. *Nature*.

[61] Hsu J., Hodgins J. J., Marathe M. (2018). Contribution of NK cells to immunotherapy mediated by PD-1/PD-L1 blockade. *Journal of Clinical Investigation*.

[62] Ansell S. M. (2019). Immunotransplant: preventing unintended consequences. *Cancer Discovery*.

[63] Kim N., Kim H. S. (2018). Targeting checkpoint receptors and molecules for therapeutic modulation of natural killer cells. *Frontiers in Immunology*.

[64] Huang Y., Chen Z., Jang J. H. (2018). PD-1 blocks lytic granule polarization with concomitant impairment of integrin outside-in signaling in the natural killer cell immunological synapse. *Journal of Allergy and Clinical Immunology*.

[65] Pesce S., Greppi M., Tabellini G. (2017). Identification of a subset of human natural killer cells expressing high levels of programmed death 1: a phenotypic and functional characterization. *Journal of Allergy and Clinical Immunology*.

[66] Concha-Benavente F., Kansy B., Moskovitz J., Moy J., Chandran U., Ferris R. L. (2018). PD-L1 mediates dysfunction in activated PD-1(+) NK cells in head and neck cancer patients. *Cancer Immunology Research*.

[67] Vey N., Karlin L., Sadot-Lebouvier S. (2018). A phase 1 study of lirilumab (antibody against killer immunoglobulin-like receptor antibody KIR2D; IPH2102) in patients with solid tumors and hematologic malignancies. *Oncotarget*.

[68] Chiossone L., Dumas P. Y., Vienne M., Vivier E. (2018). Natural killer cells and other innate lymphoid cells in cancer. *Nature Reviews Immunology*.

[69] Solomon B. L., Garrido-Laguna I. (2018). TIGIT: a novel immunotherapy target moving from bench to bedside. *Cancer Immunology, Immunotherapy*.

[70] He Y., Peng H., Sun R. (2017). Contribution of inhibitory receptor TIGIT to NK cell education. *Journal of Autoimmunity*.

[71] Zhang B., Zhao W., Li H. (2016). Immunoreceptor TIGIT inhibits the cytotoxicity of human cytokine-induced killer cells by interacting with CD155. *Cancer Immunology, Immunotherapy*.

[72] Yu X., Harden K., C Gonzalez L. (2009). The surface protein TIGIT suppresses T cell activation by promoting the generation of mature immunoregulatory dendritic cells. *Nature Immunology*.

[73] Martinet L., Smyth M. J. (2015). Balancing natural killer cell activation through paired receptors. *Nature Reviews Immunology*.

[74] Chan C. J., Martinet L., Gilfillan S. (2014). The receptors CD96 and CD226 oppose each other in the regulation of natural killer cell functions. *Nature Immunology*.

[75] Blake S. J., Dougall W. C., Miles J. J., Teng M. W. L., Smyth M. J. (2016). Molecular pathways: targeting CD96 and TIGIT for cancer immunotherapy. *Clinical Cancer Research*.

[76] Zhang Q., Bi J., Zheng X. (2018). Blockade of the checkpoint receptor TIGIT prevents NK cell exhaustion and elicits potent anti-tumor immunity. *Nature Immunology*.

[77] Xu F., Sunderland A., Zhou Y., Schulick R. D., Edil B. H., Zhu Y. (2017). Blockade of CD112R and TIGIT signaling sensitizes human natural killer cell functions. *Cancer Immunology, Immunotherapy*.

[78] Parham P., Guethlein L. A. (2018). Genetics of natural killer cells in human health, disease, and survival. *Annual Review of Immunology*.

[79] Sun C., Xu J., Huang Q. (2017). High NKG2A expression contributes to NK cell exhaustion and predicts a poor prognosis of patients with liver cancer. *Oncoimmunology*.

[80] Platonova S., Cherfils-Vicini J., Damotte D. (2011). Profound coordinated alterations of intratumoral NK cell phenotype and function in lung carcinoma. *Cancer Research*.

[81] McWilliams E. M., Mele J. M., Cheney C. (2016). Therapeutic CD94/NKG2A blockade improves natural killer cell dysfunction in chronic lymphocytic leukemia. *Oncoimmunology*.

[82] Ruggeri L., Urbani E., Andre P. (2016). Effects of anti-NKG2A antibody administration on leukemia and normal hematopoietic cells. *Haematologica*.

[83] Muntasell A., Ochoa M. C., Cordeiro L. (2017). Targeting NK-cell checkpoints for cancer immunotherapy. *Current Opinion in Immunology*.

[84] Sarkar S., van Gelder M., Noort W. (2015). Optimal selection of natural killer cells to kill myeloma: the role of HLA-E and NKG2A. *Cancer Immunology, Immunotherapy*.

[85] Delconte R. B., Kolesnik T. B., Dagley L. F. (2016). CIS is a potent checkpoint in NK cell-mediated tumor immunity. *Nature Immunology*.

[86] Molgora M., Supino D., Mantovani A., Garlanda C. (2018). Tuning inflammation and immunity by the negative regulators IL-1R2 and IL-1R8. *Immunological Reviews*.

[87] Molgora M., Bonavita E., Ponzetta A. (2017). IL-1R8 is a checkpoint in NK cells regulating anti-tumour and anti-viral activity. *Nature*.

[88] Mamessier E., Sylvain A., Thibult M. L. (2011). Human breast cancer cells enhance self tolerance by promoting evasion from NK cell antitumor immunity. *Journal of Clinical Investigation*.

[89] Rusakiewicz S., Semeraro M., Sarabi M. (2013). Immune infiltrates are prognostic factors in localized gastrointestinal stromal tumors. *Cancer Research*.

[90] Navarro A. G., Björklund A. T., Chekenya M. (2015). Therapeutic potential and challenges of natural killer cells in treatment of solid tumors. *Frontiers in Immunology*.

[91] Alkins R., Burgess A., Kerbel R., Wels W. S., Hynynen K. (2016). Early treatment of HER2-amplified brain tumors with targeted NK-92 cells and focused ultrasound improves survival. *Neuro-Oncology*.

[92] Wu L., Zhang F., Wei Z. (2018). Magnetic delivery of Fe3O4@polydopamine nanoparticle-loaded natural killer cells suggest a promising anticancer treatment. *Biomaterials Science*.

[93] Park W., Gordon A. C., Cho S. (2017). Immunomodulatory magnetic microspheres for augmenting tumor-specific infiltration of natural killer (NK) cells. *ACS Applied Materials & Interfaces*.

[94] Keydar Y., le Saux G., Pandey A. (2018). Natural killer cells’ immune response requires a minimal nanoscale distribution of activating antigens. *Nanoscale*.

[95] Huang B., Abraham W. D., Zheng Y., Bustamante López S. C., Luo S. S., Irvine D. J. (2015). Active targeting of chemotherapy to disseminated tumors using nanoparticle-carrying T cells. *Science Translational Medicine*.

[96] Xue J., Zhao Z., Zhang L. (2017). Neutrophil-mediated anticancer drug delivery for suppression of postoperative malignant glioma recurrence. *Nature Nanotechnology*.

[97] Wu M., Zhang H., Tie C. (2018). MR imaging tracking of inflammation-activatable engineered neutrophils for targeted therapy of surgically treated glioma. *Nature Communications*.

[98] Siegler E. L., Kim Y. J., Chen X. (2017). Combination cancer therapy using chimeric antigen receptor-engineered natural killer cells as drug carriers. *Molecular Therapy*.

[99] Mitchell M. J., King M. R. (2014). Leukocytes as carriers for targeted cancer drug delivery. *Expert Opinion on Drug Delivery*.

[100] Pitchaimani A., Nguyen T. D. T., Aryal S. (2018). Natural killer cell membrane infused biomimetic liposomes for targeted tumor therapy. *Biomaterials*.

[101] Deng G., Sun Z., Li S. (2018). Cell-membrane immunotherapy based on natural killer cell membrane coated nanoparticles for the effective inhibition of primary and abscopal tumor growth. *ACS Nano*.

[102] Chen X., Han J., Chu J. (2016). A combinational therapy of EGFR-CAR NK cells and oncolytic herpes simplex virus 1 for breast cancer brain metastases. *Oncotarget*.

[103] Suryadevara C. M., Riccione K. A., Sampson J. H. (2016). Immunotherapy gone viral: bortezomib and oHSV enhance antitumor NK-cell activity. *Clinical Cancer Research*.

[104] Kang T., Huang Y., Zhu Q. (2018). Necroptotic cancer cells-mimicry nanovaccine boosts anti-tumor immunity with tailored immune-stimulatory modality. *Biomaterials*.

[105] Loftus C., Saeed M., Davis D. M., Dunlop I. E. (2018). Activation of human natural killer cells by graphene oxide-templated antibody nanoclusters. *Nano Letters*.

[106] Ben-Akiva E., Meyer R. A., Wilson D. R., Green J. J. (2017). Surface engineering for lymphocyte programming. *Advanced Drug Delivery Reviews*.

[107] Nakamura T., Yamada K., Fujiwara Y., Sato Y., Harashima H. (2018). Reducing the cytotoxicity of lipid nanoparticles associated with a fusogenic cationic lipid in a natural killer cell line by introducing a polycation-based siRNA core. *Molecular Pharmaceutics*.

[108] Reiners K. S., Kessler J., Sauer M. (2013). Rescue of impaired NK cell activity in Hodgkin lymphoma with bispecific antibodies *in vitro* and in patients. *Molecular Therapy*.

[109] Vallera D. A., Felices M., McElmurry R. (2016). IL15 trispecific killer engagers (TriKE) make natural killer cells specific to CD33+ targets while also inducing persistence, in vivo expansion, and enhanced function. *Clinical Cancer Research*.

[110] Tavri S., Jha P., Meier R. (2009). Optical imaging of cellular immunotherapy against prostate cancer. *Molecular Imaging*.

[111] Lim Y. T., Cho M. Y., Noh Y.-W., Chung J. W., Chung B. H. (2009). Near-infrared emitting fluorescent nanocrystals-labeled natural killer cells as a platform technology for the optical imaging of immunotherapeutic cells-based cancer therapy. *Nanotechnology*.

[112] Meier R., Piert M., Piontek G. (2008). Tracking of [^18^F]FDG-labeled natural killer cells to HER2/neu-positive tumors. *Nuclear Medicine and Biology*.

[113] Mallett C. L., Mcfadden C., Chen Y., Foster P. J. (2012). Migration of iron-labeled KHYG-1 natural killer cells to subcutaneous tumors in nude mice, as detected by magnetic resonance imaging. *Cytotherapy*.

[114] Jang E. S., Shin J. H., Ren G. (2012). The manipulation of natural killer cells to target tumor sites using magnetic nanoparticles. *Biomaterials*.

[115] Riley J. L. (2013). Combination checkpoint blockade--taking melanoma immunotherapy to the next level. *New England Journal of Medicine*.

[116] Li T., Pan S., Gao S. (2019). Diselenide-pemetrexed assemblies for combined cancer immuno-, radio-, and chemotherapies. *Angewandte Chemie International Edition*.

[117] Fillon M. (2018). Immune checkpoint inhibitors are superior to docetaxel as second-line therapy for patients with non-small cell lung carcinoma. *CA: A Cancer Journal for Clinicians*.

[118] Rosenberg S. A., Restifo N. P. (2015). Adoptive cell transfer as personalized immunotherapy for human cancer. *Science*.

[119] Keating S. E., Zaiatz-Bittencourt V., Loftus R. M. (2016). Metabolic reprogramming supports IFN-*γ* production by CD56brightNK cells. *The Journal of Immunology*.

[120] Assmann N., O'Brien K. L., Donnelly R. P. (2017). Srebp-controlled glucose metabolism is essential for NK cell functional responses. *Nature Immunology*.

[121] Loftus R. M., Assmann N., Kedia-Mehta N. (2018). Amino acid-dependent cMyc expression is essential for NK cell metabolic and functional responses in mice. *Nature Communications*.

[122] Zou Y., Li F., Hou W., Sampath P., Zhang Y., Thorne S. H. (2014). Manipulating the expression of chemokine receptors enhances delivery and activity of cytokine-induced killer cells. *British Journal of Cancer*.

[123] Ivanova D., Krempels R., Ryfe J., Weitzman K., Stephenson D., Gigley J. P. (2014). NK cells in mucosal defense against infection. *BioMed Research International*.

[124] Klingemann H. (2014). Are natural killer cells superior CAR drivers?. *Oncoimmunology*.

